# Acquired and hereditary bone marrow failure: A mitochondrial perspective

**DOI:** 10.3389/fonc.2022.1048746

**Published:** 2022-11-02

**Authors:** Waseem Nasr, Marie-Dominique Filippi

**Affiliations:** ^1^ Division of Experimental Hematology and Cancer Biology, Cincinnati Children’s Research Foundation, Cincinnati, OH, United States; ^2^ University of Cincinnati College of Medicine, Cincinnati, OH, United States

**Keywords:** bone marrow failure (BMF), mitochondria, TGF beta, innate immune signaling, myelodysplastic disorder (MDS)

## Abstract

The disorders known as bone marrow failure syndromes (BMFS) are life-threatening disorders characterized by absence of one or more hematopoietic lineages in the peripheral blood. Myelodysplastic syndromes (MDS) are now considered BMF disorders with associated cellular dysplasia. BMFs and MDS are caused by decreased fitness of hematopoietic stem cells (HSC) and poor hematopoiesis. BMF and MDS can occur *de novo* or secondary to hematopoietic stress, including following bone marrow transplantation or myeloablative therapy. *De novo* BMF and MDS are usually associated with specific genetic mutations. Genes that are commonly mutated in BMF/MDS are in DNA repair pathways, epigenetic regulators, heme synthesis. Despite known and common gene mutations, BMF and MDS are very heterogenous in nature and non-genetic factors contribute to disease phenotype. Inflammation is commonly found in BMF and MDS, and contribute to ineffective hematopoiesis. Another common feature of BMF and MDS, albeit less known, is abnormal mitochondrial functions. Mitochondria are the power house of the cells. Beyond energy producing machinery, mitochondrial communicate with the rest of the cells *via* triggering stress signaling pathways and by releasing numerous metabolite intermediates. As a result, mitochondria play significant roles in chromatin regulation and innate immune signaling pathways. The main goal of this review is to investigate BMF processes, with a focus mitochondria-mediated signaling in acquired and inherited BMF.

## Introduction

Ineffective hematopoiesis leading to the absence of one or more hematopoietic lineages in the peripheral blood represents broad and heterogeneous blood disorders comprised of bone marrow failure (BMF) and myelodysplastic (MDS) syndromes. Patients with BMF or MDS suffer from a severe reduction of one or more hematopoietic lineages in the peripheral blood, which is life-threatening (1–4) BMF can be inherited or acquired. The most common inherited BMFs include Fanconi anemia, Shwachman-Diamond syndrome, congenital amegakaryocytic thrombocytopenia, and reticular dysgenesis. Other inherited BMF are X-linked recessive dyskeratosis congenita and Blackfan-Diamond Anemia (5–7). MDS, which are now classified as acquired disorders that resemble BMF with a variety of cell dysplastic features, may occur *de novo* or secondary to BMF. MDS are classified in several groups based on established clinical and histopathological features, as defined by the World Health Organization: MDS with single lineage dysplasia, MDS with ring sideroblasts (MDS-RS), MDS with multilineage dysplasia, MDS with excess blasts (MDS-EB), MDS with isolated del(5q). BMF/MDS can also appear after allogenic or autologous hematopoietic stem cell transplantation (HSCT) (8, 9), as well as after myeloablative chemotherapy (10), and are called therapy-related BMF/MDS. Some patients develop secondary MDS/AML within 6 years of autologous HSCT (11). Although BMF/MDS are very heterogeneous, the same genomic mutations are frequently found in MDS patients such as mutations in genes related to RNA splicing (SF3B1, SRSF2, U2F1, ZRSR2), DNA methylation (TET2, DNMT3A, IDH1/IDH2), chromatin modification (ASXL1, EZH2), transcription regulation (RUNX1, BCOR), and DNA repair control (p53). These observations suggest that additional environmental factors largely contribute to disease development. Substantial clinical data have shown that hyperactivity of inflammatory cytokines, including TNFα, IL-6, and transforming growth factor–β (TGFβ), directly contribute to hematopoietic failure in BMF/MDS.1 (12–14), Chronic inflammation and enhanced innate immune signaling are also recognized as contributing factors of inefficient hematopoiesis (15–17). Interestingly, several evidence suggest that disruption of mitochondria is another preponderant factor in BMF/MDS development. Abnormal mitochondria have been linked to both acquired and hereditary BMF (18–24). Patients with MDS have transcriptional, morphological and functional dysregulation of their mitochondria, according to several studies (25–28). Some of these defects are the direct consequences of abnormal expression of nuclear-encoded mitochondrial genes. Others could arise in response to stress or the inflammatory milieu. The functions of mitochondria, which supply energy and metabolic activity in response to cellular demand (29), go well beyond energy production. Mitochondria communicate with the rest of the cell through activation of signaling pathways and control a broad range of cellular functions such as apoptosis, iron metabolism and heme production. In addition, mitochondria participate in the generation of metabolite intermediates, acetyl-CoA and S-Adenosyl-Methionine (SAM), used for epigenetic remodeling, as well as those for *de novo* biosynthetic processes, including nucleotides and fatty acids. Finally, mitochondria control the cellular response to stress, including inflammation stress. This review will discuss the emerging role of mitochondria as driver of *de novo* or secondary BMF/MDS. It will discuss the potential mechanism, direct or indirect, of how abnormal mitochondrial functions contribute to ineffective hematopoiesis.

## MDS with sideroblasts are mitochondrial diseases

### Mitochondria are home of heme synthesis

The first step in heme biosynthesis takes place into mitochondria and involves the condensation of succinyl-CoA and glycine to form δ-aminolevulinic acid (ALA) in the mitochondrial matrix. This reaction is catalyzed by ALA synthase (ALAS). There are two isoforms of ALAS, ALAS1 and ALAS2, which is found exclusively in erythroid cells. ALA is exported to the cytosol *via* SLC25A38 and ABCB10 where it is converted to coproporphyrinogen III (CPgenIII). CPgenIII is imported back into mitochondria, where it is converted to protoporphyrinogen IX by coproporphyrinogen oxidase (CPOX). Then, protoporphyrinogen IX is oxidized to protoporphyrin IX (PPIX) by protoporphyrinogen oxidase (PPOX). Finally, ferrous iron is incorporated into PPIX to form heme in the mitochondrial matrix, a reaction catalyzed by ferrochelatase (FECH) (30). T the expression of both Alas2 and *FECH* is controlled by iron, thus linking the regulation of heme biosynthesis in erythroid cells to the availability of iron. Iron is acquired by differentiating erythroid progenitors *via* transferrin receptor 1 (TfR1)-mediated endocytosis and transferred to mitochondria for heme synthesis *via* mitoferrin1 (*MFRN1*) and mitoferrin2 (*MFRN2*), expressed in erythroid and non-erythroid tissues, respectively. The generation of globin and heme levels in erythroid precursors is balanced by a cell membrane heme exporter known as feline leukemia virus subgroup C receptor 1 (FLVCR1). Flvcr1b, an isoform of Flvcr1 that is present in mitochondria, facilitates heme efflux into the cytoplasm (31).

### MDS with RS: A mitochondrial disorder affecting the erythroid lineage

Sideroblastic anemia, congenital or acquired, are associated with MDS and are characterized by the presence of ring sideroblasts, which result from decreased heme production and excess iron deposit within mitochondria of erythroid cells (32). Mutations in genes related to heme synthesis are drivers of MDS-RS. Mutation in ALAS2 reduces protoporphyrin causing an accumulation of iron in mitochondria and subsequently cell death. Mutations in Ala carriers, *ABCB7 and* in Slc25a38, also reduces heme synthesis and causes MDS-RS. Germline mutation in the Glutaredoxin 5 [*GLRX5]* gene causes iron overload and is associated with sideroblastic-like microcytic anaemia. GLRX5 is a mitochondrial protein, which is involved in the biogenesis of iron-sulfur clusters.

The splicing factor SF3B1 is the most commonly mutated genes in MDS with the disease phenotype with ring sideroblasts (33, 34). SF3B1 Splicing factor 3b, together with splicing factor 3a and a 12S RNA unit, forms the U2 small nuclear ribonucleoproteins complex (U2 snRNP) and binds pre-mRNA upstream of the intron’s branch site in a sequence independent manner. SF3B1 mutations cause abnormal mitochondrial iron absorption and ineffective erythropoiesis (35). Initial studies found that SF3B1-mutant erythroblasts displayed larger quantities of mitochondria. When co-mutation with EZH2 occurs, mitochondrial membrane potential is abnormal and ROS are increased; likely driving cell death (1). The exact molecular mechanism behind the abnormal mitochondrial functions and iron deposition is being uncovered. Mutated-SF3B1 notably targets expression of genes involved in mitochondrial heme synthesis such as *PPOX*, *TMEM14C and Abcb7*, causing a block in protoporphyrin synthesis (36–39). Interestingly, SFB1 mutation confers proliferation advantage to the clone. In a remarkable study by Hsu (40), the clonal evolution of MDS was studied using iPSCs reprogrammed from patient samples and shows that the initial mutation is t (4, 12), followed by mutations in SF3B1, EZH2, and del(5q), in that sequence.

Another study described the importance of the Retinoblastoma protein (pRb) gene, a crucial cell cycle regulator that controls the transition from the G1 to the S phase, in mitochondrial functions in erythroid cells. Deletion of Rb in erythroid cells caused poor erythropoiesis with dysplastic features due to abnormal mitochondrial biogenesis and cell cycle exit. Erythroid-specific deletion of pRb led to decreased expression of mitochondria-related genes, a reduction in mitochondrial membrane potential and a change in the ROS produced by the mitochondria. Expression of critical oxidative phosphorylation genes such Ndufa1 (complex 1, OXPHOS), Atp5s (ATP synthesis), and Cox7b (electron transfer), expression of the mitochondrial biogenesis gene PGC1b, of the mitochondrial antioxidant Prdx3, which is crucial for maintaining the balance of (ROS), as well as ALAS2, and ABCB were all decreased. In this Rb-deficiency mouse model, overexpressing PGC1b was sufficient to normalize the RBC counts, underscoring the critical role of mitochondria in the pathogenesis of the disease (27).

**Figure 1:**
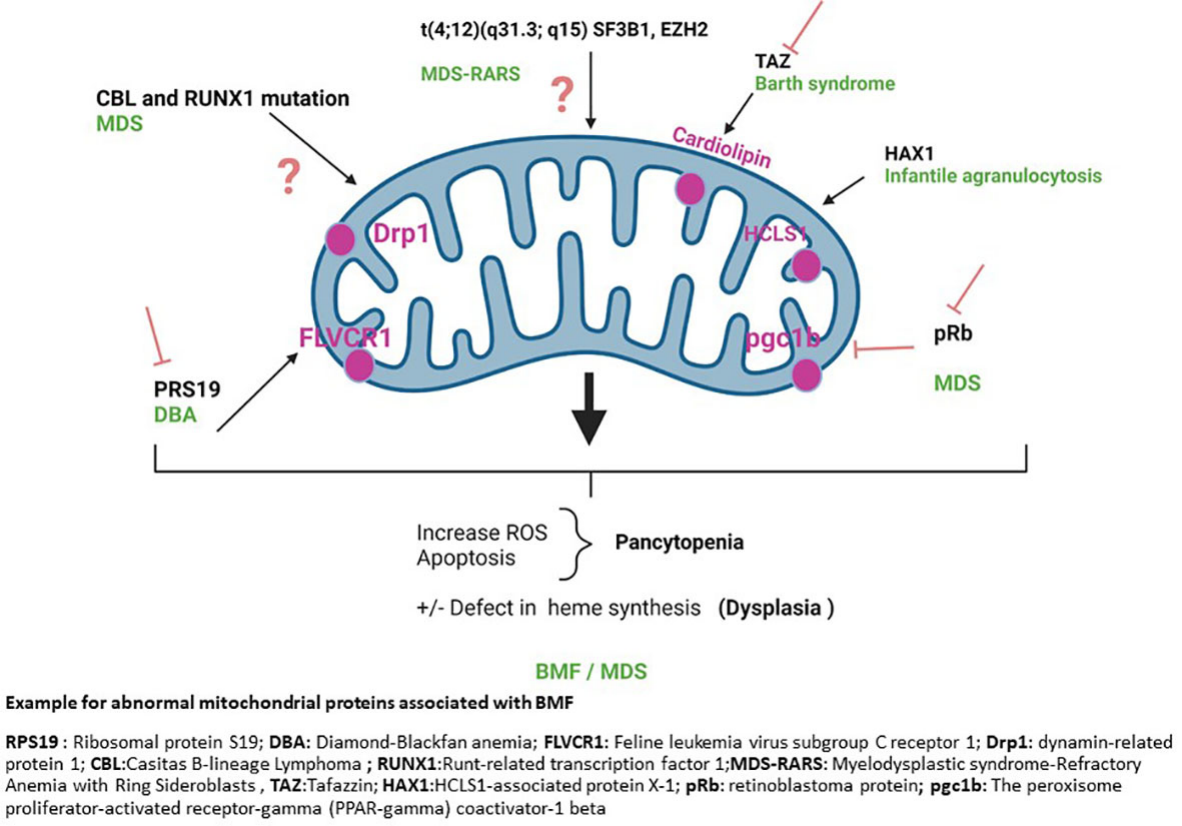
Example for abnormal mitochondrial proteins associated with BMF. RPS19, Ribosomal protein S19; DBA, Diamond-Blackfan anemia; FLVCR1, Feline leukemia virus subgroup C receptor 1; Drp1, dynamin-related protein 1; CBL, Casitas B-lineage Lymphoma; RUNX1, Runt-related transcription factor 1; MDS-RARS, Myelodysplastic syndrome-Refractory Anemia with Ring Sideroblasts, TAZ, Tafazzin; HAX1, HCLS1-associated protein X-1; pRb, retinoblastoma protein; pgc1b, The peroxisome proliferator-activated receptor-gamma (PPAR-gamma) coactivator-1 beta.

Finally, decreased FLVCR1 levels or increased expression of aberrant alternative splicing of FLVCR1 transcript are seen in DBA patients and a cellular model of DBA (41, 42). Downregulation of FLVCR1a and FLVCR1b results in an increase in oxidative stress, cell cycle arrest at G0/G1, and apoptosis due to heme accumulation. This is yet another illustration of how a flaw in mitochondrial homeostasis can result in ineffective erythropoiesis and BMF. Germline mutations in other genes, such as *PUS1*, *YARS2*, *SLC19A2* and *TRNT1*, as well as mitochondrial DNA deletions, have been identified in distinct forms of inherited sideroblastic anemias (32) (**Figure 1**).

## Mutations in genes that alter the generation of mitochondrially –produced metabolites cause MDS

Mitochondrial functions and epigenetic regulation are tightly linked in several ways. One way is through the tricarboxylic acid (TCA) cycle – a major mitochondrial metabolic pathway. The TCA cycle produces several intermediate metabolites, citrate, alpha-ketoglutarate (α-KG), itaconate, succinate, fumarate, malate and oxaloacetate, through a series of enzymatic reactions. When the TCA metabolites are coupled with the mitochondrial electron transport chain, TCA intermediates are used for subsequent metabolic reactions through oxidative phosphorylation (OXPHOS) to generate ATP. TCA metabolites also serve in non-metabolic signaling roles. For example, itaconate, succinate, fumarate have all been shown to alter the innate immune response. In addition, the TCA metabolites are directly involved in epigenetic regulation. Succinate and fumarate can directly inhibit the activity of histone or DNA demethylase. Alpha-KG is needed for the activity of DNA demethylase. Acetyl-CoA serve as donor group of histone acetylation; S-Adenosyl-Methionine (SAM) which serve as donor group for DNA or histone methylation is generated through a complex interaction between the mitochondrial one-carbon folate pathway and the methionine cycle (43). The tight connection between mitochondrial metabolism and chromatin regulation is one component of the preponderant, yet ill-understood, role of mitochondria in MDS pathogenesis. Mutations in the TCA enzyme IDHs (*IDH1-IDH2)* occur in about 7% of MDS cases, with *IDH2* mutations being more frequent (about 4.5%) than *IDH1* mutations (about 2.5%).  (44) *IDH2* mutations are particularly enriched in the RAEB subtype of MDS. IDH1/2 catalyzes the oxidation of isocitrate to oxalosuccinate within the TCA cycle, which is followed by decarboxylation of the carboxyl group beta to the ketone to form α-KG. This reaction also generates NADPH. 44 Mutations in IDH1/2 thus by impacting α-KG production alter the activity of metabolic enzymes that depend on α-KG availability, such as the DNA demethylase Tet2. Tet2 primarily catalyzes the oxidation of 5-methylcytosine (5mC) to 5-hydroxymethylcytosine (5hmC). Mutations in Tet2 are also associated with clonal hematopoiesis, increased risk of MDS progression, and poor prognosis in AML (45). Interestingly,* *IDH2* mutations* are mutually exclusive with *TET2* and *SF3B1* mutations, but are frequently associated with *SRSF2* mutations. Other known mutations in epigenetic regulators that are associated with clonal hematopoiesis and MDS are found in the DNA methylase DNMT3a (46). DNMT3A, catalyzes the methylation of CpG dinucleotides in genomic DNA, which is dependent on SAM availability. Hence, stressors that alter mitochondrial functions could easily contribute to disease development and participate in disease heterogeneity in a given genetic background. Any abnormality in mitochondrial functions that would cause abnormal production of SAM or aKG would also alter Tet2 or DNMT3a functions and could drive MDS pathogenesis without the need for somatic mutation in specific genes (see below).

Because of the link between mitochondria and epigenetics, altered mitochondrial metabolism is a common characteristics of MDS/BMF that drives disease phenotype. Interestingly, in addition to exhibiting specific metabolic alterations that result from the genetic context, MDS have a common abnormal metabolic signature. Elegant studies from the Huang’s lab showed that hypoxia-inducible factor 1α (HIF1A) transcriptional signature is generally activated in MDS patient bone marrow stem/progenitors, in major MDS-associated mutations (*Dnmt3a, Tet2, Asxl1, Runx1*, and *Mll1*). 48 Remarkably, using inducible activation of HIF1A signaling mouse model, they show that HIF1A is sufficient to induce dysplastic and cytopenic MDS phenotypes. On the other hand, both genetic and chemical inhibition of HIF1A signaling rescues MDS phenotypes in a mouse model of MDS, indicating that elevated HIF1A is necessary for MDS phenotype. Therefore, metabolic changes associated with HIF1A are central pathobiologic mediators of MDS. Two other important observations were that HIF1A signature is also associated with enrichment in several inflammatory/immune response–related pathways. Plus, it renders a state of pseudohypoxia and mitochondrial dysfunction in which expressions of nuclear-encoded mitochondrial genes, notably the electron transport chain complex II that is normally important for OXPHOS, are downregulated. In this context, metabolites of the TCA cycle, aKG, succinate, fumarate and malate, accumulate – thus further altering cellular functions (47).

## Abnormal mitochondrial dynamics contributes to MDS

Mitochondria are very dynamic organelles, whose numbers and organization can vary greatly. (48–50) The mitochondrial network can be organized into interconnected and fused filaments, or into fragmented and smaller unit (48). Mitochondrial fusion is controlled by mitofusins 1 and 2 (Mfn1 and Mfn2) and optic atrophy 1 and 3 (Opa1, Opa3). Mitochondrial fission is regulated by dynamin-related protein 1 (Drp1) and fission protein 1 (Fis1) (48–50). Mitochondrial dynamism is important to adapt cells to energy demand. When energy demand is high, mitochondria are fused and mitochondrial oxidative phosphorylation [OXPHOS] is favored. Mitochondrial fusion also enables ‘mixing’ mitochondrial membrane proteins for repair mechanisms. A recent study describes the role of aberrant mitochondrial fission as driver of MDS. They found a MDS patient harboring a mutation in both the E3 ubiquitin-protein ligase CBL gene and the transcription factor RUNX1 gene. In a mouse model of *CBL* exon deletion with RUNX1 mutants, that recapitulated clinically relevant MDS phenotypes, HSC and progenitor cells exhibited excessive mitochondrial fragmentation that was caused by enhanced activity of the mitochondrial fission regulator DRP1. The subsequent elevation in ROS production and inflammatory signals promoted the development of dysplasia and impaired granulopoiesis (18). Other studies have reported abnormal mitochondrial structure, or abnormal mitochondrial biogenesis and mitophagy in BMF, notably in FA. In this case, they found that FA genes are required for selective autophagy, which removes unwanted cytoplasmic contents including mitochondria such that FA gene deficiency results in impaired virophagy and antiviral host defense, decreased Parkin-mediated mitophagy, and increased mitochondrial ROS-dependent inflammasome activation (51). Loss of FA-gene-mediated selective autophagy may contribute to the pathophysiology of FA-gene associated diseases.

## Role of reactive oxygen species in developing BMF/MDS

One common characteristic of BMF/MDS is the higher susceptibility of myeloid progenitors to apoptosis. Excessive myeloid cell apoptosis contributes to peripheral cytopenias even when the bone marrow is hypercellular. Numerous variables, including internal or external reactive oxygen species (ROS), can cause apoptosis. ROS comprise radical and non-radical molecules (52), and are often released as a byproduct of oxidative phosphorylation or during mitochondrial stress conditions. To counteract ROS, HSC express enzymatic and nonenzymatic defensive mechanisms, such as superoxide dismutase, glutathione peroxidase, myeloperoxidase, Vitamins C, E, and reduced glutathione (GSH) (53). When compared to controls, MDS patients have significantly higher levels of intracellular peroxides in lymphocytes, erythroid precursors, monocytes and granulocytes, as well as a considerably lower superoxide/peroxides ratio and GSH levels, resulting in oxidative stress and subsequent macromolecule and organelle damages (52, 54).

Chronic oxidative stress is also found in FA cells due to increased DNA damage. This is associated with mitochondrial damage and OXPHOS dysfunction. In fact, spontaneous mitochondrial fragmentation occurs in FA cells that leads to change in mitochondrial distribution, shape, and integrity (55, 56). HSPCs deficient in the FA protein Fancd2 rely on increased mitochondrial translation for survival and proliferation (57). The changes in mitochondrial structure are also accompanied by changes in metabolism. FA cells exhibit lower OXPHOS, increased glycolytic flux and decreased glutaminolysis (23). We know that a balance between glycolysis and OXPHOS is necessary for HSPC differentiation. Quiescent HSC rely mostly on anaerobic glycolysis and lysosomal functions for their energy needs. HSC activation and commitment to differentiation are associated with increased aerobic glycolysis, mitochondrial activation and increased OXPHOS. Hence, abnormal mitochondrial function in FA cells could substantially impact the ability of HSPC to differentiate, further contributing the FA pathogenesis  (23). Similarly, a study focused on SBDS gene that affects ribosome biogenesis, mitotic spindle assembly, chemotaxis, and ROS generation, shows that lower expression of SBDS causes defective mitochondria and elevated ROS (58) (**Figure 1**).

## Other mitochondrial abnormalities linked to inherited BMF

Mitochondrial abnormalities are found in a variety of hematologic phenotypes. The Pearson syndrome (24, 59), is a multisystem mitochondrial respiratory chain disorder caused by a single large scale mitochondrial DNA deletion. Patients present with pancytopenia sideroblastic anemia and exocrine pancreatic insufficiency. The Barth syndrome, which presents with neutropenia in addition to musculoskeletal defects and cardiomyopathy, is another mitochondrial disorder (22, 60).The Barth syndrome is caused by a mutation in the gene TAZ. TAZ encodes for the mitochondrial phospholipid transacylase tafazzin. TAZ controls the production of tetralinoleoyl cardiolipin, a mitochondrial membrane-specific lipid. When tafazzin is knocked down by shRNA in mice, tetralinoleoyl cardiolipin levels are drastically reduced, and monolysocardiolipins accumulate in mitochondria (61).The aberrant buildup of monolysocardiolipins causes mitochondrial dysfunction (62) leading to enhanced mitochondrial membrane potential breakdown, abnormal cytochrome c release, caspase-3 activation (63) and cellular death, including in myeloid precursors and neutrophils (64). Similarly, in the Kostmann disease also known as infantile agranulocytosis (severe congenital neutropenia) nonsense mutations in mitochondrial-associated antiapoptotic protein (HCLS1) lead to premature stop codons, loss of function, and frequently, total loss of protein expression, causing acute neutropenia that is often accompanied by neurologic and cognition impairments (65, 66). Finally, BMF and MDS have been linked to mutations in mitochondrial DNA (67) (**Figure 1**).

It has long been known that mitochondria can be taken up by cells and can transfer from cell to cell, *in vivo* or *in vitro*. Because of this, mitochondrial replacement or supplementation in cells is being proposed as novel therapeutic approach of mitochondrial diseases. A recent study shows that mitochondrial augmentation in human CD34+ cells from healthy donors or patients with mitochondrial DNA disorder can prove beneficial (68). The group described a method of ex vivo transfer of HSPC with normal exogenous mitochondria, that they termed mitochondrial augmentation therapy (MAT). They show that MAT can improve mitochondrial content and oxygen consumption of healthy and diseased HSPCs. Importantly, they used xenotransplant in immunodeficient NSGS mice to show that MAT confers HSPCs from a patient with an mtDNA disorder superior human engraftment (68).

## Secondary BMF/MDS following hematopoietic stress from bone marrow transplantation: Importance of abnormal mitochondrial functions

Secondary MDS and AML are becoming more widely known as late complications of stem cell transplantation (69). The incidence of treatment-related MDS and AML is between 5 percent and 20 percent 5-10 years after an autologous stem cell transplantation (ASCT) (10). Among all cancers, MDS development occurred in 35% of non-Hodgkin’s lymphoma patients who receive ASCT (70). In a Japanese study, 1.38% lymphoma patients receiving ASCT developed secondary myeloid dysplasia 3 years after transplant; 0.37% lymphoma patients receiving allogeneic SCT developed secondary MDS 3 years later (71). The causes and mechanisms behind the development of secondary BMF/MDS are multiple and could be cell intrinsic or arising from damage in the bone marrow microenvironment.

One clear cell intrinsic mechanism is linked to the *therapy* of the original disorder. There is strong evidence that alkylating anti-leukemic drugs, or whole body irradiation (TBI) used pre-transplantation cause chromosomal damage that can result in MDS/AML (72).

*Increased inflammation* following treatment could also be a contributing factor. Inflammatory factors are altered after BMT. Among those factors is the transforming growth factor beta (TGFβ). TGFβ is known to suppress cellular growth and to contribute to ineffective hematopoiesis (73–77). The allogeneic and autologous stem cell transplantation conditioning protocols decrease TGFβ production (78, 79). After roughly 7 weeks of bone marrow repopulation, the plasma level of TGFβ returns to normal (80, 81). Interestingly, this is seen in mouse model as well. Research from our lab has demonstrated that while TGFβ levels are lower in the bone marrow microenvironment, TGFβ protein and signaling are enhanced in HSPC after bone marrow transplantation. In this context, TGFβ acts through p38MAPK to impair HSC self-renewal and cause ineffective hematopoiesis after bone marrow transplantation (13). Increased inflammatory cytokines and associated inflammatory signals are a common characteristic of BMD/BMF. TGFβ plasma levels are elevated in hematopoietic cells of patients with myelodysplastic syndromes MDS (82–84). TGFβ signaling is also elevated in FA patients (85, 86). In a Fanca-deficient mouse model, challenge with polyinosinic:polycytidylic acid (pIC) leads to changing DNA repairing system *via* enhanced TGFβ signaling and causes BMF due to increased DNA mutations (75). TGFβ signaling inhibition restored hematopoiesis in this mouse model. TGFβ is also elevated in Shwachman-Bodian-Diamond Condition (SBDS) and Diamond Blackfan anemia (DBA) 2. Blocking the TGFβ pathway using a small molecule inhibitor or a TGF-family ligand trap can ameliorate the inefficient erythropoiesis also found in SDS or Diamond Blackfan anemia patients (87–89).

Overall, the role of inflammation in BMF or MDS development is now established. Toll-like receptors (TLRs) or their signaling effectors are often overexpressed in MDS samples compared to healthy controls, enhancing a type I interferon response through NFkB, MAPK, and IRF3 (12, 90, 91). The inflammasome is elevated in BMF/MDS patients and contributes to ineffective hematopoiesis (92–94). The inflammasome is a multiprotein complex composed of the sensor of damage associated molecular patterns (DAMPs), ie NLRP3 Nod-like receptor, an adaptor protein apoptosis-associated speck-like protein containing a caspase recruitment domain (ASC), and caspase-1 causing the release of interleukin-1b (IL-1b) and IL-18 and cell death by pyroptosis (95). Inflammatory milieu and cell death it creates contribute to pancytopenia and ineffective hematopoiesis. In addition, inflammation could provide a selective advantage to mutated HSC clones, as seen in a model of Dnmt3a-loss of functions in which chronic infection drives clonal expansion of the Dnmt3a-mutant clones *via* INFy (96). Therefore, increased inflammation and/or inflammatory cytokines following transplantation or hematopoietic stress could be a factor contributing to secondary BMF/MDS.

*Viral infections* activate DNA and RNA sensing pathways to trigger innate immune signaling pathways that converge on an interferon response. The DNA-sensing pathway, cGAS/STING, activates NFkB, IRF3 to clear the viral infection. In response to viral RNA, the innate immune response starts with cytosolic viral RNA sensor retinoic acid inducible gene-I (RIG-I). Then, RIG-I engages the adaptor protein MAVS (Mitochondrial AntiViral Signaling). In turn, MAVS, which is anchored onto mitochondria, triggers a sequence of signaling that converge onto NFkB, IRF3 or the inflammasome (97–99). These pathways have been involved in MDS, directly or indirectly. DDX41 can activate cGAS/STING; mutation in DDX41 are associated with MDS (100). It is important to note that numerous viruses have been connected to the formation of MDS, including CMV (101), HTLV-1 (102), parvovirus B19 (103), and HHV-6 (104). In a very interesting retrospective study on lymphoma patients who developed secondary AML following HSCT indicates that at the time of stem cell transplantation, 1% of patients who received auto-SCT and 5% of patients who received allo-SCT had infections (71). The hypothesis that MDS could start as a viral infection was suggested more than 20 years ago (105). The infection could trigger dysregulated cytokine production in the BM microenvironment, providing optimal growth support to stem cells harboring a mutation (8, 9, 106, 107)., as recent studies are now demonstrating (96).

*Stress-induced abnormal mitochondria*. Interestingly, abnormal expression of nuclear-encoded mitochondrial genes is a predicting factor of therapy-related MDS (108). Consistent with this, we have shown that HSC keep abnormal mitochondria after BMT, indicating that the stress of BMT permanently alters mitochondria and HSC functions (109). The mechanisms causing alteration in mitochondrial functions in HSC following transplantation involves deregulation in mitochondrial dynamism and decreased expression of mitochondrial genes (109). Inability to remove abnormal mitochondria could contribute to secondary BMF/MDS in multiple ways. Abnormal mitochondria likely cause abnormal metabolism, including abnormal TCA cycle and OXPHOS that could mimic IDH or Tet2 mutation phenotypes. Abnormal mitochondria could contribute to secondary BMF/MDS *via* activation of innate immune signaling. Indeed, mitochondria serve as platform of innate immune signaling. Activation of numerous innate immune pathways occurs at the plasma membrane of mitochondria and depends on mitochondrial regulation (110, 111). For instance, activation of the inflammasome can depend on ROS production from stressed mitochondria (112). Viral infections, as seen above, lead to MAVS activation (97–99). MAVS activation requires mitochondrial polarization (i.e., established mitochondria membrane potential [MMP]) and is enhanced by mitochondrial fusion (111). Conversely, termination of the innate immune response is mediated by removal of mitochondria *via* mitophagy (113–115). Finally, mitochondrial stress is often accompanied by an abnormal release of mitochondrial DNA, which could activate DNA sensing pathways and subsequently innate immune signaling. Hence, abnormal mitochondria could be a mediator of inflammation following transplantation directly or in the context of added infection, and thus create an inflammatory a context for a mutated clone to expand.

**Figure 2:**
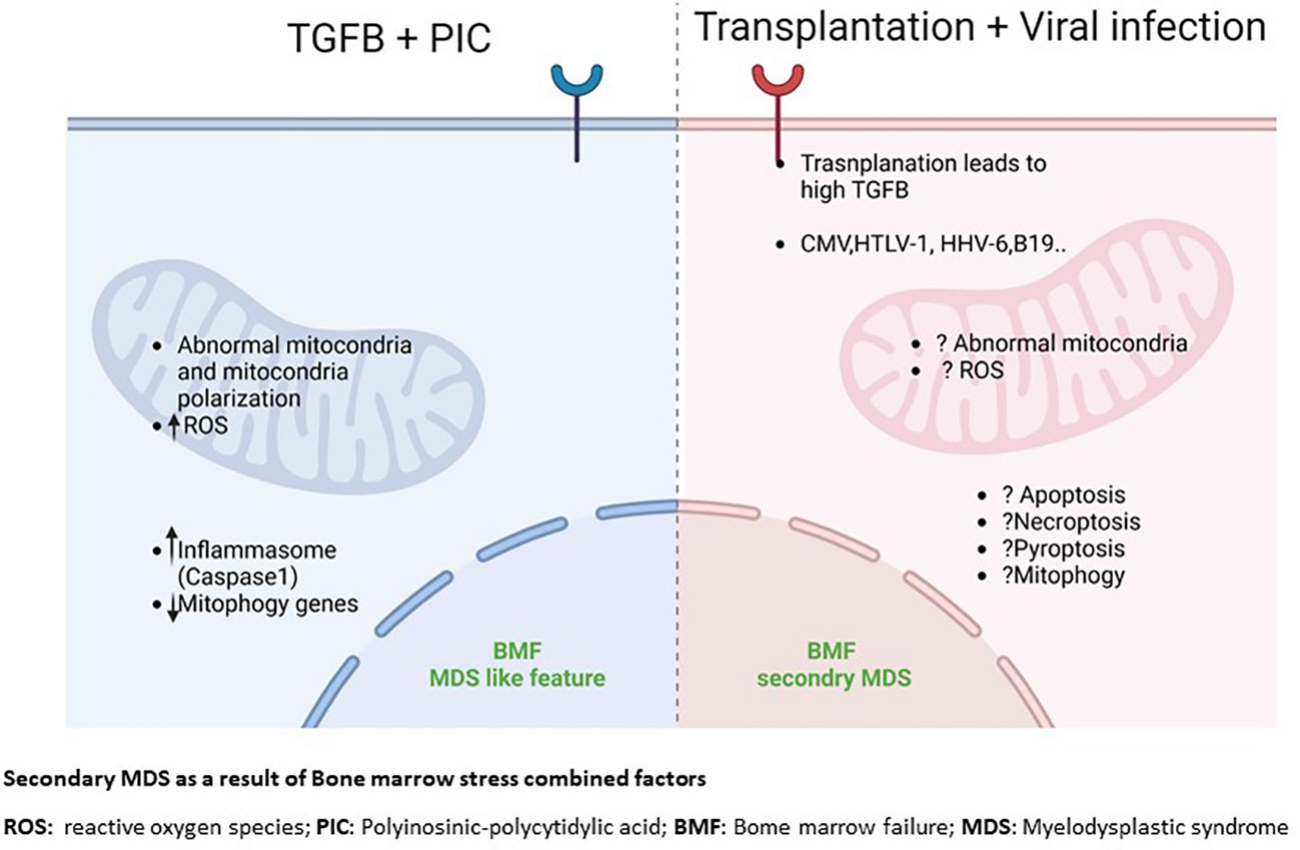
Secondary MDS as a result of Bone marrow stress combined factors. ROS, reactive oxygen species; PIC, Polyinosinic-polycytidylic acid; BMF, Bome marrow failure; MDS, Myelodysplactic syndrome.

*The case for combinatorial effects: possible interactions between dysregulated TGFB, defective mitochondria and innate immune pathways as causal factors of secondary BMF/MDS.* TGFβ upregulation and mitochondria abnormality occur in tandem in many BMFs. The source or consequence of this relationship is not completely clear. Our group recently reported the possible link between overexpression of TGFβ and mitochondria in the development of BMF/MDS (106). Using an TGFβ overexpressing mouse model, we demonstrated that elevated TGFβ signaling alone is not sufficient to cause BMF or MDS. However, elevated TGFβ signaling plus challenge with the double-stranded RNA pIC cause chronic pancytopenia, bone marrow dysplasia, increased hematopoietic stem and progenitor cell pools, which are phenotypes to human BMF. We further showed that elevated TGFβ plus pIC challenge alters mitochondrial functions with an elevated mitochondrial membrane potential and mitochondrial content. The gene expression profile of HSC shows persistent changes in the transcription profile in HSC from overexpressed TGFβ mice challenged with pIC that includes nuclear-encoded mitochondrial genes. Only overexpressed TGFβ HSC had higher expression of mrpl46 and other genes essential for the regulation of mitochondrial translation following pIC stress. This phenotype was associated with elevated levels of reactive oxygen species, and caspase-1 activation (106). Our findings imply that bone marrow failure with dysplastic features can occur without a prior genetic damage when chronic enhanced TGFβ signaling changes the acute immune response to pIC. Because pIC triggers an innate immune response mimicking a viral response, TGFβ may alter the innate immune pathways by modifying mitochondrial response, thus leading to development of an environment favored for BMF/MDS initiation and progression. These findings suggest a combinatorial effect between TGFβ and mitochondrial-mediated innate immune pathways could contribute to secondary BMF/MDS (**Figure 2**).

## Conclusions and future directions

The formation of BMF involves numerous crucial interrelated factors, including genetics, proteomics, nutrition, cellular signaling, metabolism, and interaction between the HSC and other stromal cells. Mitochondria are emerging as important factors in the pathogenesis of BMF/MDS. How exactly mitochondria contribute to BMF/MDS remain to be analyzed in detail. We need to further understand the potential effects of damaged mitochondria on BMF/MDS development, including the potential consequences on HSC metabolism in disease context and how metabolic changes contribute to disease development. Examining this will need to be done both in the context of *de novo* BMF/MDS and secondary BMF/MDS. How the stress of bone marrow transplantation, with or without viral infection, alters mitochondrial functions is another area of interest. The fact that mitochondria serve as platform of innate immune signaling is very intriguing and will need to be examined in detail, as abnormal mitochondria could represent an important mechanism of hyperinflammation associated with BMF/MDS. Finally, the development of mitochondrial transfer or metabolic reprogramming through metabolite addition could be complimentary to current therapeutics, and need to be carefully evaluated. Therefore, a fuller understanding of interplay between mitochondrial functions and inflammation is essential for both our fundamental understanding of HSC biology and BMF/MDS pathogenesis as well as for the developmental of novel therapies. It will be important to systematically investigate the role of mitochondrial functions and associated metabolism in BMF/MDS.

## Author contributions

WN and M-DF equally contributed to writing the manuscript. M-DF edited the manuscript. All authors contributed to the article and approved the submitted version.

## Funding

This work was supported by the Department of Defense award BM 190093 to M-DF.

## Conflict of interest

The authors declare that the research was conducted in the absence of any commercial or financial relationships that could be construed as a potential conflict of interest.

## Publisher’s note

All claims expressed in this article are solely those of the authors and do not necessarily represent those of their affiliated organizations, or those of the publisher, the editors and the reviewers. Any product that may be evaluated in this article, or claim that may be made by its manufacturer, is not guaranteed or endorsed by the publisher.
